# Toxicity of Food-Grade TiO_2_ to Commensal Intestinal and Transient Food-Borne Bacteria: New Insights Using Nano-SIMS and Synchrotron UV Fluorescence Imaging

**DOI:** 10.3389/fmicb.2018.00794

**Published:** 2018-04-24

**Authors:** Joanna M. Radziwill-Bienkowska, Pauline Talbot, Jasper B. J. Kamphuis, Véronique Robert, Christel Cartier, Isabelle Fourquaux, Esther Lentzen, Jean-Nicolas Audinot, Frédéric Jamme, Matthieu Réfrégiers, Jacek K. Bardowski, Philippe Langella, Magdalena Kowalczyk, Eric Houdeau, Muriel Thomas, Muriel Mercier-Bonin

**Affiliations:** ^1^Institute of Biochemistry and Biophysics, Polish Academy of Sciences, Warsaw, Poland; ^2^Micalis Institute, INRA, AgroParisTech, Université Paris-Saclay, Jouy-en-Josas, France; ^3^Toxalim (Research Centre in Food Toxicology), Université de Toulouse, INRA, ENVT, INP-Purpan, UPS, Toulouse, France; ^4^Faculté de Médecine Rangueil, Centre de Microscopie Electronique Appliquée à la Biologie (CMEAB), Toulouse, France; ^5^Luxembourg Institute of Science and Technology (LIST), Material Research and Technology Department (MRT), Belvaux, Luxembourg; ^6^Synchrotron SOLEIL, Gif-sur-Yvette, France

**Keywords:** food-grade TiO_2_, bacterial toxicity, intestinal bacteria, food-borne bacteria, cellular and subcellular bioimaging

## Abstract

Titanium dioxide (TiO_2_) is commonly used as a food additive (E171 in the EU) for its whitening and opacifying properties. However, a risk of intestinal barrier disruption, including dysbiosis of the gut microbiota, is increasingly suspected because of the presence of a nano-sized fraction in this additive. We hypothesized that food-grade E171 and Aeroxyde P25 (identical to the NM-105 OECD reference nanomaterial in the European Union Joint Research Centre) interact with both commensal intestinal bacteria and transient food-borne bacteria under non-UV-irradiated conditions. Based on differences in their physicochemical properties, we expect a difference in their respective effects. To test these hypotheses, we chose a panel of eight Gram-positive/Gram-negative bacterial strains, isolated from different biotopes and belonging to the species *Escherichia coli*, *Lactobacillus rhamnosus*, *Lactococcus lactis* (subsp. *lactis* and *cremoris*), *Streptococcus thermophilus*, and *Lactobacillus sakei*. Bacterial cells were exposed to food-grade E171 vs. P25 *in vitro* and the interactions were explored with innovative (nano)imaging methods. The ability of bacteria to trap TiO_2_ was demonstrated using synchrotron UV fluorescence imaging with single cell resolution. Subsequent alterations in the growth profiles were shown, notably for the transient food-borne *L. lactis* and the commensal intestinal *E. coli* in contact with food-grade TiO_2_. However, for both species, the reduction in cell cultivability remained moderate, and the morphological and ultrastructural damages, observed with electron microscopy, were restricted to a small number of cells. *E. coli* exposed to food-grade TiO_2_ showed some internalization of TiO_2_ (7% of cells), observed with high-resolution nano-secondary ion mass spectrometry (Nano-SIMS) chemical imaging. Taken together, these data show that E171 may be trapped by commensal and transient food-borne bacteria within the gut. In return, it may induce some physiological alterations in the most sensitive species, with a putative impact on gut microbiota composition and functioning, especially after chronic exposure.

## Introduction

Engineered nanomaterials are increasingly used in numerous industrial sectors, due to their unique properties compared to their larger counterparts, provided by their nanometric dimensions and their high specific surface area (up to several hundred m^2^/g of product) (Nel et al., 2006). Among nanomaterials, titanium dioxide (TiO_2_) is widely used, notably in the food industry, as a white coloring agent (referred to as food-grade additive E171 in the EU) for confectionery, sauces, cakes, and pastries. In the United States, the Food and Drug Administration approved the use of food-grade TiO_2_ in 1966 with the stipulation that TiO_2_ should not exceed 1% by weight of the food (Joint Fao/Who Expert Committee on Food Additives, 2006). In Europe, EU Directive 94/36/EC authorizes the use of E171 in processed food, without establishment of an acceptable daily intake by the Joint FAO/WHO Expert Committee on Food Additives, because intestinal TiO_2_ absorption was considered very low (Efsa Panel on Food Additives and Nutrient Sources Added to Food, 2005). A sizable fraction (17–55% of total particles) of nano-sized particles (<100 nm) is produced during the manufacturing process of the powder, depending on the commercial supplier of the E171 (Weir et al., 2012; Yang et al., 2014; Bettini et al., 2017; Dorier et al., 2017). The presence of this nano-sized fraction is increasingly suspected to play a role in intestinal barrier disruption, including dysbiosis of the gut microbiota. Daily dietary intake has been estimated to be 1–2 mg TiO_2_/kg body weight (bw) for US children under 10 years of age, and 0.2–0.7 mg TiO_2_/kg bw for others (Weir et al., 2012). In Europe, estimated daily exposure levels have ranged between 0.2 and 0.4 mg/kg bw in infants and the elderly, and 5.5 and 10.4 mg/kg bw in children, depending on the exposure scenario (Efsa Panel on Food Additives and Nutrient Sources Added to Food, 2016). Recent studies report deleterious effects of E171 on the gut epithelial barrier *in vitro* (Faust et al., 2014; Dorier et al., 2017; Proquin et al., 2017), some of which possibly predispose the host to intestinal diseases and colorectal cancer, as shown in rodents (Urrutia-Ortega et al., 2016; Bettini et al., 2017).

Even though an increasing body of evidence suggests that the gut microbiota is a major player in food toxicology (Claus et al., 2016; Ribière et al., 2016; Jin et al., 2017), the interactions of dietary nanoparticles with commensal intestinal and/or transient food-borne bacteria are largely unknown (Fröhlich and Fröhlich, 2016; Fröhlich and Roblegg, 2016; Mercier-Bonin et al., 2016; Pietroiusti et al., 2016). The impact of E171 on the composition and metabolic activity of the gut microbiota has recently been assessed *in vitro* (Dudefoi et al., 2017a; Waller et al., 2017). However, most of the literature to date focuses on the TiO_2_ photocatalytic antibacterial applications under UV light (McCullagh et al., 2007; Liu et al., 2010; Pigeot-Rémy et al., 2011; Carré et al., 2014; Joost et al., 2015), even though increasing attention is being paid to bacterial inactivation in the absence of light. Here, *Escherichia coli* was generally chosen as the bacterial model (Liu et al., 2010; Kumar et al., 2011; Zhukova et al., 2012; Erdem et al., 2015; Sohm et al., 2015; Planchon et al., 2017), using standard nano-sized particles (TiO_2_-NPs) (Simon-Deckers et al., 2009; Liu et al., 2010; Kumar et al., 2011; Pagnout et al., 2012; Zhukova et al., 2012; Erdem et al., 2015; Sohm et al., 2015; Planchon et al., 2017), despite their physicochemical properties differing significantly from those of food-grade TiO_2_ (Yang et al., 2014; Dudefoi et al., 2017b).

Based on this background, the present study focuses on probing interactions between food-grade TiO_2_ (E171) and several representative commensal intestinal and transient food-borne bacteria under non-UV irradiated conditions, by coupling different innovative (nano)imaging methods. Results were compared with those of the NM-105 (TiO_2_-NPs P25) OECD reference nanomaterial. To this end, the TiO_2_ trapping properties of bacteria were evaluated using Synchrotron excitation deep ultraviolet (DUV) fluorescence imaging with single-cell resolution. Concurrently, the TiO_2_-mediated effects on bacterial growth were assessed. To gain further insight into the putative TiO_2_-driven toxicity to bacteria, morphological changes after exposure were evaluated with electron microscopy (SEM and TEM), in combination with high-resolution nano-secondary ion mass spectrometry (Nano-SIMS) to determine TiO_2_ internalization.

## Materials and Methods

### Particle Preparation

The E171 sample was obtained from a French commercial supplier of food coloring. P25 (NM-105) nanomaterial was provided by the European Union Joint Research Centre (EU JRC) as a test material of manufactured TiO_2_ nanoparticles (Aeroxyde P25) and selected by the Organization for Economic Cooperation and Development (OECD) for safety evaluation of titanium-based nanomaterials. It displays mixed crystallinity with anatase as the predominant form (85% anatase: 15% rutile), and a mean particle diameter of 22 ± 1 nm (Rasmussen et al., 2014). The TiO_2_ products, in their metrological aggregated (A) and dispersed (D) forms, were prepared according to the generic Nanogenotox dispersion protocol depicted by Jensen et al. (2011). Briefly, a 2.56 mg/mL stock suspension was prepared by pre-wetting the powder in absolute ethanol, followed by dispersion in 0.05% (w/v) BSA solution and probe sonication on ice for 27 min at 40% amplitude (Sonifier Cell Disruptor Model 250 20 kHz, Branson Ultrasonics Corporation). The stock suspensions were always prepared fresh prior to each experiment, sonicated if required, and diluted using MilliQ-grade water to the target test concentrations (0–320 μg/mL). The physicochemical characterization of the TiO_2_ particles has been detailed elsewhere (Talbot et al., submitted). Spectrofluorimetric analysis was performed using a FluoroMax-4 (HORIBA Jobin Yvon, Inc., Chilly-Mazarin, France) spectrofluorometer. Measurements were performed using 10-mm path length quartz cuvettes. Fluorescence emission spectra of food-grade E171 vs. TiO_2_-NPs P25 were recorded at 20°C at an excitation wavelength of 270 nm.

### Bacterial Strains and Culture Conditions

Eight Gram-positive/Gram-negative commensal intestinal or food-borne bacterial strains, isolated from different biotopes and belonging to the species *Escherichia coli*, *Lactobacillus rhamnosus*, *Lactococcus lactis* (subsp. *lactis* and *cremoris*), *Streptococcus thermophilus*, and *Lactobacillus sakei* were used in this study (**Table 1**). Growth kinetics experiments were performed for food-grade E171 and TiO_2_-NPs P25 in batch cultures under non-UV irradiated conditions, with an early addition of TiO_2_ (i.e., addition of TiO_2_ to the culture medium before cell growth). Culture conditions are given in **Table 1**. For all strains, a first inoculum was obtained by adding 0.1 mL of stock culture into 5 mL of culture medium, followed by 6 h of incubation under the appropriate culture conditions (**Table 1**). A volume of 0.1 mL of this preculture was then used to inoculate 10 mL of culture medium. This second preculture was incubated overnight under the same conditions until the stationary phase of growth, and then used to inoculate cultures to evaluate the effect induced by TiO_2_. A high-throughput method based on sterile microwell plates (384 wells; Greiner, Bio-one) was implemented. From the culture of each strain grown overnight, the optical density at 600 nm (OD_600nm_) was adjusted to 0.025 in appropriate culture medium. The aliquots of 70 μL from this OD_600nm_-adjusted culture were deposited into wells (at least 5 wells per condition). Then, 10 μL of TiO_2_ particles (E171 vs. P25), in the aggregated (A) or dispersed (D) metrological form, were added to achieve an exposure concentration of 320, 125, 62.5, or 32 μg/mL. Wells without TiO_2_ particles and with only TiO_2_ particles in appropriate culture medium were used as control and blank, respectively. Growth of the TiO_2_-exposed and non-exposed strains was then monitored every 15 min for 24 h by measuring the OD_600nm_ (with simultaneous stirring) using a dark-controlled and thermoregulated (30 or 37°C depending on the strain under study) plate reader (Infinite M200 PRO, Tecan France). The strain tolerance to TiO_2_ stress was assessed by determining the duration of the lag phase (h), the maximal specific growth rate μ_max_ (h^-1^), and the final OD_600nm_ for each condition. Experiments were performed at least in duplicate on independently grown cultures to validate data reproducibility. In parallel, cell viability was evaluated by spreading-plating TiO_2_-exposed (test concentration of 320 μg/mL) and non-exposed bacteria on appropriate agar medium. Plates were incubated for 24 h at the appropriate temperature and the number of developed colony-forming units (CFU) was counted and expressed as CFU/mL. The viability percentage of the bacteria after TiO_2_ exposure was calculated by dividing their CFU (CFU/mL) by that in the TiO_2_-free control.

**Table 1:** List of bacterial strains under study.

Species	Strain	Gram characteristics	Origin	Culture conditions
*Escherichia coli*	K12 MG1655	Gram-negative	Laboratory strain	LB medium, 37°C with shaking
	ATCC8739		Commensal, isolated from infant feces	
	CEC15		Commensal, isolated from a conventional suckling rat intestine	
*Lactobacillus rhamnosus*	GG	Gram-positive	Commensal, isolated from a healthy human intestine	MRS medium, 37°C without shaking
*Lactobacillus sakei*	23K	Gram-positive	Food (meat)	MRS medium, 30°C without shaking
*Lactococcus lactis* subsp. *cremoris*	IBB477	Gram-positive	Food (raw milk)	M17 medium supplemented with glucose 0.5% (m/v), 30°C without shaking
*Lactococcus lactis* subsp. *lactis*	TIL448		Food (peas)	
*Streptococcus thermophilus*	LMD-9	Gram-positive	Food (yogurt)	M17 medium supplemented with lactose 1% (m/v), 37°C without shaking


### Synchrotron Excitation Deep Ultraviolet (DUV) Fluorescence Imaging of Bacterial Cells After TiO_2_ Exposure

Deep ultraviolet fluorescence imaging of *E. coli* K12 MG1655 and *L. lactis* IBB477 cells after TiO_2_ exposure was carried out on a Zeiss Axio Observer Z-1 microscope at the DISCO beamline (Giuliani et al., 2009) of the SOLEIL synchrotron facility, as previously described for tryptophan-functionalized gold (Pajović et al., 2015) and silver nanoparticles (Dojčilović et al., 2016). Bacterial cells were cultured in appropriate medium containing TiO_2_ at a concentration of 320 μg/mL (aggregated vs. dispersed forms, E171 vs. P25), collected at the stationary phase of growth and washed twice in phosphate buffered saline (5000 g, 5 min, room temperature). Thereafter, 2 μL of the washed cell suspension were placed on quartz coverslips (ESCO Optics, United States) and dried in ambient conditions for 30 min. Bacteria were first observed in bright field through a 100× Zeiss Ultrafluar objective with a 1.25 numerical aperture that requires glycerin immersion. Afterwards, the samples were illuminated by a 270-nm monochromatized synchrotron beam, which was used as the excitation source. The fluorescent signals were collected by a PIXIS 1024 BUV camera (Princeton, United States) in the spectral ranges 327–353 nm (Filter I, OMEGA Filters United States) and 420–480 nm (Filter II, OMEGA Filters, United States) during 90 s of integration time. In addition, to increase the signal to noise ratio, images were recorded with binning of the pixels (2 × 2) in μManager software (Edelstein et al., 2010), used to control the whole setup. For each condition, two independent quartz coverslips with at least three different locations, covering a minimum of 200 bacterial cells, were investigated to validate the consistency of the observations. The images were analyzed using FIJI software (ImageJ, NIH) (Schindelin et al., 2012). A set of specific FIJI macro scripts was developed to standardize the analysis (see Supplementary Material 1).

### Electron Microscopy of *E. coli* and *L. lactis* Bacterial Cells Subjected to TiO_2_

For scanning electron microscopy (SEM) observations, TiO_2_-exposed (test concentration of 320 μg/mL) and non-exposed *E. coli* K12 MG1655 and *L. lactis* IBB477 bacterial cells were harvested by centrifugation (5000 g, 5 min, room temperature), washed twice with phosphate-buffered saline, fixed and colored with a 2.5% glutaraldehyde + 0.04% Ruthenium red solution in sodium cacodylate buffer (0.1 M, pH = 7.4) for at least 4 h at 4°C. Cells were made to adhere on poly-lysine coated coverslips and washed in distilled water prior to dehydration in graded ethanol series. Desiccation was carried out in a Leica EM CPD300 automatic critical point drier. Samples were then mounted on stubs and coated with 10 nm platinum (Leica EM MED020). Examinations were carried out on a FEI Quanta FEG 250 scanning electron microscope at a 5 kV accelerating voltage.

For transmission electron microscopy (TEM), the TiO_2_-exposed and non-exposed cells were washed, fixed, colored as described above, then embedded in 2% Low Melting Point Agarose and washed in sodium cacodylate buffer (0.2 M) with 0.04% Ruthenium red. The post-fixation step was carried out in a mixture solution of osmium tetroxide (1%) and 0.04% Ruthenium red in sodium cacodylate buffer (0.2 M) for 1 h at room temperature. Cell dehydration was achieved through a series of graded ethanol solutions up to 70%. Infiltration was performed in an automatic microwave tissue processor Leica EM AMW, using ethanol, then acetone and several changes of Embed 812 resin/acetone mixtures, in which the concentration of resin was gradually increased to 100%. Cells were then embedded in Embed 812 (EMS) and left to polymerize 48 h at 60°C. Polymerized samples were sectioned in 90-nm-thick slices (Ultracut Reichert) and mounted on 150 mesh collodion-coated copper grids. Before observation, sections were stained with uranyl acetate and lead citrate. TEM observations were carried out on a Hitachi HT7700 microscope, at an accelerating voltage of 80 kV.

### Elemental Composition Mapmaking of *E. coli* and *L. lactis* Bacterial Cells Subjected to TiO_2_

Polymerized samples, obtained as described above, were sectioned in 300-nm thick slices (Ultracut Reichert) and mounted on silicon plots (Siltronix, Archamps, France). Elemental maps were obtained by Nano-SIMS using a NanoSIMS50 instrument (Cameca, Gennevilliers, France) (Höschen et al., 2015). The surface was scanned as a matrix of 256 × 256 pixels for an area of 10 μm^2^ by an energetic primary cesium ion beam with an acceleration of 8 kV and a primary current of 1.2 pA. The secondary negative ions emitted with 8 kV were filtered in mass, detected and counted simultaneously, allowing an elemental mapping of the original voxel sputtered (Georgantzopoulou et al., 2013). In these conditions, the pixel size was about 40 nm for an estimated probe size of 100 nm. The ions recorded were ^12^C^14^N, ^32^S, ^31^P^16^O_2_, ^46^Ti^16^O, and ^48^Ti^16^O with a mass resolution M/ΔM higher than 5000. The Ti element was mapped (as TiO^-^ cluster) on at least 100 individual cells of each sample. The distribution of the ^12^C^14^N^-^ cluster, well known by the SIMS community, was simultaneously recorded as a “fingerprint” of bacterial cells. Due to the probability of ionization and the matrix effect, i.e., large variations in the ionization yields, SIMS is not a direct quantitative technique (Behrisch and Wittmaack, 1991). However, by normalization of the trace element by the matrix element signals, a quantitative analysis (coupled with a reference sample) or a semi-quantitative analysis can be performed with an excellent accuracy (Dowsett and Collins, 1996). Unfortunately, for biological samples, it is very difficult to obtain standard samples. This explains why only the semi-quantitative approach was proposed here, based on the normalization of the signal of the titanium cluster (TiO) by the signal intensity of the main ion detected, the cluster (CN). The intensity ratio (TiO/CN) was then calculated for each cell and for all conditions (Audinot et al., 2004; Biesemeier et al., 2016). A total number between 200 and 300 cells was considered (taking into account the aggregated and dispersed TiO_2_ forms). The signal intensities of the different elements were extracted from image acquisitions using OpenMIMS, a FIJI plugin developed at Harvard at the National Resource for Imaging Mass Spectrometry^1^ (McMahon et al., 2006).

### Statistical Analysis

Replicates in this study were technical and biological, i.e., repeated experiments with the same and independently grown bacterial cultures, respectively. Significance was determined using one-way ANOVA corrected for multiple comparisons with Dunnett test or two-way ANOVA corrected for multiple comparisons with Bonferroni test (GraphPad Prism software, version 4). Significance was set at ^∗^*p* < 0.05.

## Results

### DUV Fluorescence Imaging of Growing *L. lactis* and *E. coli* Cells Exposed to TiO_2_

**FIGURE 1:**
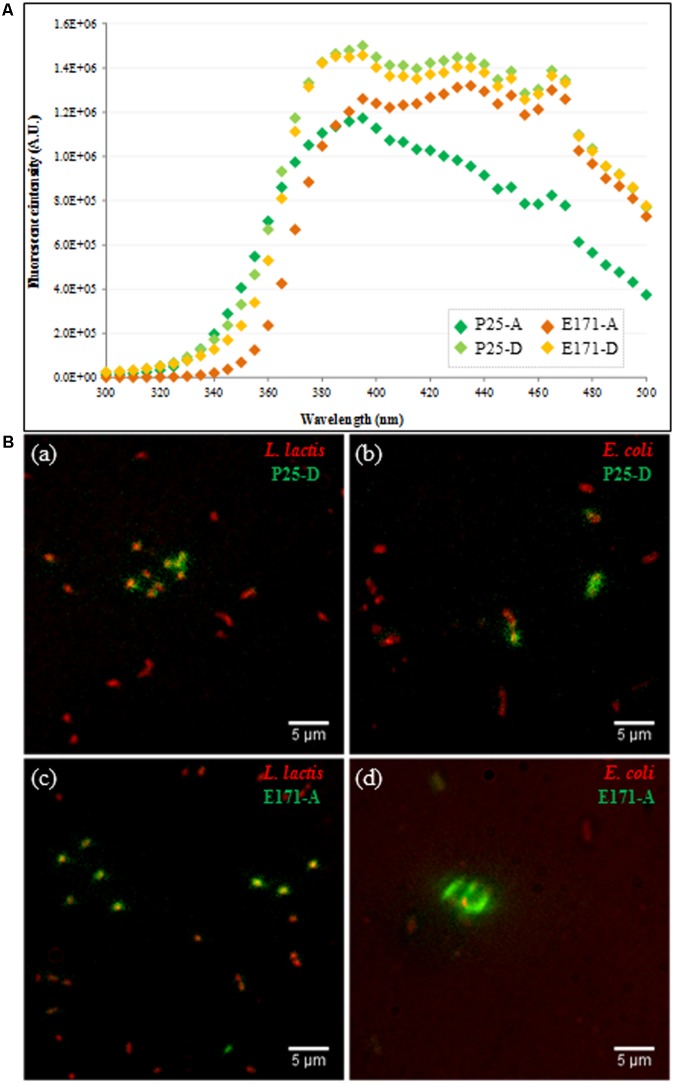
Synchrotron excitation deep ultraviolet (DUV) fluorescence imaging of bacterial cells after Titanium dioxide (TiO_2)_ exposure. **(A)** Fluorescence emission spectra (λ_exc_ = 270 nm) of TiO_2_ 320 μg/mL food-grade E171 (in orange) vs. TiO_2_-NPs P25 (in green) in aggregated (dark color) and dispersed (light color) forms; **(B)** Overlaid fluorescence images of **(a,c)** growing *Lactococcus lactis* IBB477 cells and **(b,d)** growing *Escherichia coli* K12 MG1655 cells (in red) exposed to **(a,b)** P25-D and **(c,d)** E171-A (in green) at 320 μg/mL. Yellow color indicates TiO_2_ particles that are co-localized with bacteria. Scale bar corresponds to 5 μm.

For this study, Gram-positive *L. lactis* (IBB477 strain) and Gram-negative *E. coli* (K12 MG1655 strain) were chosen as representatives of transient food-borne and commensal species of the gut microbiota, respectively. DUV fluorescence imaging of *L. lactis* and *E. coli* growing bacterial cells exposed to food-grade E171 vs. TiO_2_-NPs (P25) at 320 μg/mL was performed using a 270-nm synchrotron beam as excitation source to obtain a high TiO_2_/autofluorescence ratio. First, food-grade E171 particles in aggregated (A) and dispersed (D) forms, like their P25 counterparts, could be excited in the DUV range at 270 nm and displayed fluorescence emission spectra with characteristic peaks of TiO_2_ (Serpone et al., 1995), i.e., a maximum of fluorescence emission around 390 nm with fluorescence intensity remaining at high levels up to 470 nm (**Figure 1A**). Interestingly, whatever the type of TiO_2_, the maximum of the fluorescence emission increased as the size of TiO_2_ particles decreased. Next, interactions between bacteria and TiO_2_ were visualized based on the differences in fluorescence emission spectra between bacteria and TiO_2_ particles. The signals in the [327–353 nm] range originate from cell autofluorescence, mainly due to tryptophan fluorescence [peaking at around 340 nm (Jamme et al., 2010; Kašèáková et al., 2012)], which is responsible for the characteristic optical properties of many proteins (Lakowicz, 2006), whereas, as seen above (**Figure 1A**), emission from TiO_2_-exposed bacterial cells in the [420–480 nm] range is attributed to the fluorescence of TiO_2_ particles, located inside and/or on the surface of the bacterial cells. Overlays of the fluorescence images acquired within [327–353 nm] and [420–480 nm] detection ranges are given in **Figure 1B** for *L. lactis* and *E. coli* exposed to food-grade TiO_2_ (E171-A) and TiO_2_-NPs P25 (P25-D). We clearly demonstrated that DUV fluorescence imaging allows for non-invasive monitoring of TiO_2_ without any artificial labeling at single-cell resolution. In fact, whatever the TiO_2_ type, a TiO_2_/cell co-localization was observed for both *L. lactis* and *E. coli* (**Figures 1Ba–d**).

### TiO_2_ Toxicity to Growing Bacteria

**FIGURE 2:**
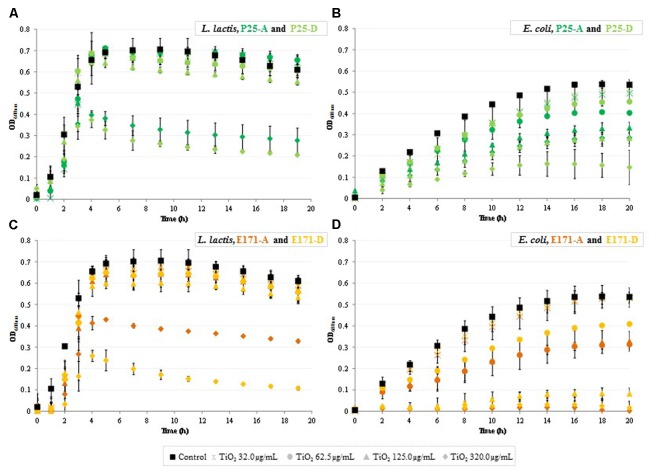
Growth kinetics of bacterial cells after TiO_2_ exposure. **(A,B)** *L. lactis* IBB477 and **(C,D)** *E. coli* K12 MG1655 subjected to 0.0 (black squares), 32.0 (crosses), 62.5 (circles), 125.0 (triangles), and 320.0 (diamonds) μg/mL TiO_2_-NPs P25 (**A,C** in green) or food-grade E171 (**B,D** in orange) in aggregated (dark color) vs. dispersed (light color) forms. Data are the means ± SD. See Supplementary Material 2 for statistical analysis.

In order to evaluate the impact of co-localization between the selected bacteria and TiO_2_ on their physiological profiles, growth kinetics were characterized in the presence of food-grade E171 or TiO_2_-NPs (P25) in the aggregated vs. dispersed forms at concentrations ranging from 32 to 320 μg/mL. These growth kinetics patterns were compared to the control condition without any TiO_2_ added. Results are displayed in **Figure 2**.

For *L. lactis* IBB477 exposed to E171 and P25, whatever the metrological form, bacterial growth was close to that of the control until a TiO_2_ concentration of 62.5 μg/mL. For the concentration of 125 μg/mL, significant alterations occurred especially for food-grade TiO_2_ in its dispersed form (**Figures 2A,B** and Supplementary Material 2) and, for the highest concentration of 320 μg/mL, growth kinetics were substantially affected under all tested conditions (*p* < 0.001, **Figures 2A,B** and Supplementary Material 2). For *E. coli* K12 MG1655, TiO_2_-induced inhibition of bacterial growth was also clearly demonstrated, with a more pronounced dose-dependent manner than for *L. lactis* (**Figures 2C,D**). In particular, for food-grade TiO_2_ at 320 μg/mL, no growth of *E. coli* occurred throughout the entire experiment, i.e., 20 h of culture (**Figure 2D**).

These effects on *L. lactis* IBB477 and *E. coli* K12 MG1655 were measured by the variations in three growth-related parameters, i.e., lag time (delay in the onset of growth), specific growth rate (μ_max_) and final biomass (OD_600nm_) between TiO_2_-exposed and non-exposed cells at the highest concentration tested (320 μg/mL). Results for E171 vs. TiO_2_-NPs P25 in the dispersed form are displayed in **Table 2**. For *L. lactis* IBB477, growth parameters were drastically affected, depending on the type of TiO_2_. For E171 and P25, respectively, lag time increased by a factor of 4.3 and 1.7, μ_max_ decreased by a factor of 2.4 and 2.3, and the final biomass was reduced by a factor of 6 and 3. After exposure of *E. coli* K12 MG1655 to P25-D, growth parameters were impacted as well. For E171-D, it was not possible to obtain quantitative data since no bacterial growth occurred at all. These deleterious effects were confirmed for other strains of the same species and were highest for food-grade TiO_2_ (**Table 2**). Among the *E. coli* strains tested, K12 MG1655 was shown to be the most sensitive. Furthermore, for the other commensal intestinal and food-borne bacteria studied (*L. rhamnosus*, *L. sakei*, *S. thermophilus*), TiO_2_-induced inhibition of bacterial growth was systematically observed [lag time: *p* < 0.01 for E171-D (*p* > 0.05 for P25-D); μ_max_ and final biomass: *p* < 0.01 for E171-D and P25-D; **Table 2**].

**Table 2:** Sensitivity of all bacterial strains to Titanium dioxide (TiO_2)_ in its dispersed form (P25-D in light green and E171-D in light orange).

		Lag time (h)		Specific growth rate μ_max_ (h^-1^)		Final biomass (OD_600nm_)	
				
Species	Strain	P25-D	E171-D	P25-D	E171-D	P25-D	E171-D
*L. lactis*	IBB477 subsp. *cremoris*	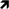 × 1.7	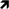 × 4.3	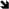 × 2.4	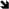 × 2.3	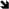 × 3.0	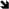 × 6.0
	TIL448 subsp. *lactis*	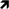 × 1.3	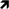 × 6.7	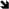 × 1.6	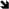 × 1.8	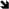 × 1.6	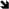 × 4.3
*L. sakei*	23K	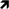 × 1.1	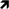 × 1.3	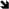 × 1.4	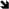 × 1.5	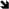 × 1.7	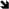 × 2.9
*S. thermophilus*	LMD-9	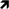 × 1.3	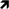 × 1.8	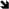 × 2.3	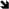 × 2.0	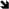 × 1.7	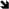 × 4.3
*L. rhamnosus*	LGG	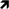 × 1.3	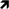 × 2.2	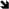 × 1.7	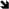 × 1.8	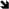 × 1.4	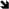 × 1.9
*E. coli*	K12 MG1655	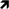 × 3.8	NA	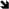 × 2.0	NA	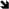 × 3.6	NA
	ATCC8739	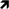 × 2.4	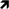 × 4.5	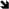 × 1.5	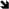 × 4.8	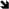 × 1.5	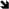 × 4.8
	CEC15	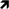 × 1.2	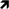 × 2.4	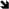 × 1.1	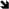 × 1.2	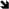 × 2.1	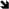 × 4.0
Significance		NS	^∗∗^	^∗∗^	^∗∗^	^∗∗^	^∗∗^


*Three growth parameters have been considered: lag time (h), specific growth rate μ_*max*_ (h^-*1*^) and final biomass (OD_*600nm*_). Ratios have been calculated with the mean values obtained for the maximal TiO_*2*_ concentration (320 μg/mL) vs. control conditions (no TiO_*2*_ added); 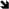 decrease, 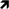 increase, NA, not available. Significance was determined for each growth parameter considering the overall P25-D- and E171-D-treated strains (except E. coli K12 MG1655) using one-way ANOVA corrected for multiple comparisons with Dunnett test: (^∗∗^) indicates p* < *0.01 compared to control conditions with no TiO_*2*_ added (NS, not significant)*.

**FIGURE 3:**
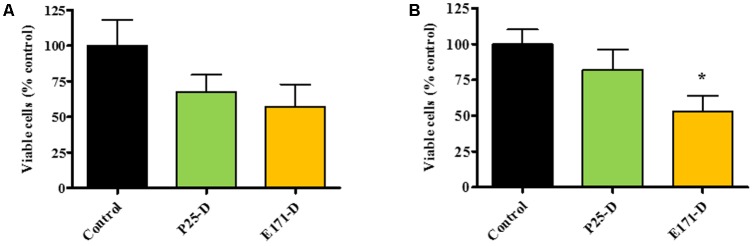
Viability of bacterial cells after TiO_2_ exposure. Viable counts (in % of the control) of *L. lactis* IBB477 **(A)** and *E. coli* K12 MG1655 **(B)** subjected to TiO_2_ at 320 μg/mL in its dispersed form D for food-grade E171 (light orange) vs. TiO_2_-NPs P25 (light green), and sampled at the end of the culture. Data are the means ± SEM. Significance was determined using one-way ANOVA corrected for multiple comparisons with Dunnett test: (^∗^) indicates *p* < 0.05 compared to control conditions with no TiO_2_ added.

The effects of TiO_2_ in its dispersed form (E171-D and P25-D) at 320 μg/mL on *L. lactis* IBB477 and *E. coli* K12 MG1655 were assessed using the plate count method for TiO_2_-exposed vs. non-exposed bacteria sampled at the end of the culture. A non-significant loss in cell cultivability was observed for *L. lactis* IBB477 (**Figure 3A**). However, for *E. coli* K12 MG1655, a moderate albeit significant reduction in viability counts (50% decrease, *p* < 0.05) occurred for food-grade TiO_2_ (**Figure 3B**). This is consistent with the above findings on the impact of TiO_2_ on the growth kinetics of *L. lactis* and *E. coli*.

### Electron Microscopy of *L. lactis* and *E. coli* Cells Exposed to TiO_2_

The interaction of *L. lactis* IBB477 and *E. coli* K12 MG1655 with TiO_2_ and the subsequent changes in cell morphology and structure were investigated with SEM and TEM.

**FIGURE 4:**
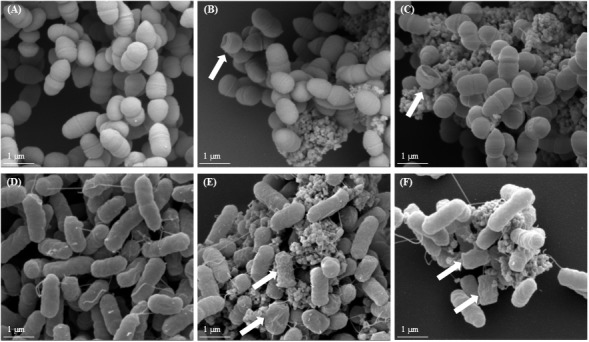
Scanning electron microscopy (SEM) images of bacterial cells after TiO_2_ exposure. **(A,D)** non-exposed growing bacterial cells, **(B,E)** growing bacterial cells exposed to E171-A 320 μg/mL, and **(C,F)** growing bacterial cells exposed to E171-D 320 μg/mL; **(A–C)** *L. lactis* IBB477, **(D–F)** *E. coli* K12 MG1655. White arrows indicate damaged bacterial cells.

**Figure 4** shows representative SEM images obtained for both species with or without exposure to food-grade E171 in its aggregated and dispersed forms at 320 μg/mL. For *L. lactis* IBB477, untreated bacteria appeared as intact cocci with no evidence of cell wall rupture and collapse (**Figure 4A**). In contrast, after TiO_2_ exposure, a small number of bacteria (see white arrows in **Figures 4B,C**) showed extensive cell wall damage and leaking out of intracellular components, probably causing cell lysis. For untreated *E. coli* K12 MG1655, the surface structure of rod-shaped bacteria was almost intact, with regular wrinkles at nano-scale resolution (**Figure 4D**). After treatment with food-grade TiO_2_, in a small number of cases, cells became twisted and rougher. Regular wrinkles were damaged and groove-like rifts appeared on the surface. The cell morphology was substantially altered, for instance cells became wider and shorter, or completely deformed (see white arrows in **Figures 4E,F**). For TiO_2_-NPs P25, the same changes were observed, although to a lesser extent (data not shown).

**FIGURE 5:**
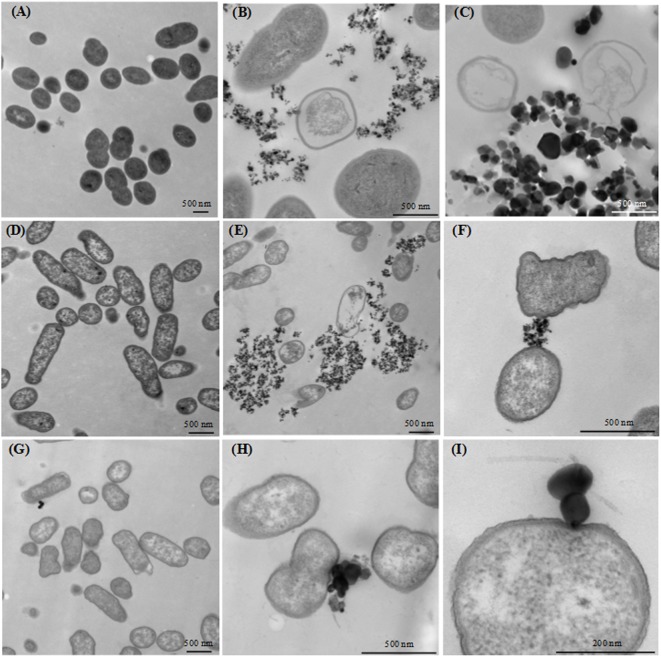
Transmission electron microscopy (TEM) images of bacterial cells after TiO_2_ exposure. **(A–C)** growing *L. lactis* IBB477 bacterial cells: **(A)** non-exposed, **(B)** exposed to P25-A 320 μg/mL and **(C)** exposed to E171-D 320 μg/mL; **(D–I)** growing *E. coli* K12 MG1655 bacterial cells: **(D)** non-exposed, **(E)** exposed to P25-A 320 μg/mL, **(F)** exposed to P25-D 320 μg/mL, **(G,H)** exposed to E171-A 320 μg/mL and **(I)** exposed to E171-D 320 μg/mL.

**Figure 5** shows TEM images of *L. lactis* IBB477 and *E. coli* K12 MG1655 bacterial cells untreated vs. treated with food-grade E171 or TiO_2_-NPs P25 in aggregated and dispersed forms at 320 μg/mL. Non-exposed bacteria remained intact with unimpaired morphology and ultrastructure, as seen in **Figure 5A,D** for *L. lactis* and *E. coli*, respectively. After TiO_2_ exposure, particle aggregates were observed attaching to the bacterial cells, but some were also present away from the cells. Of note, the primary particle size of food-grade E171 was higher than that of P25, which is consistent with previous findings (Yang et al., 2014; Bettini et al., 2017). Regarding the consequences of TiO_2_ exposure on *L. lactis* IBB477, in a few cases, cells were completely lysed and appeared as “ghosts” (**Figures 5B,C**). In some *E. coli* K12 MG1655 cells, the TiO_2_ treatment induced cell distortion, plasmolysis, and cell wall and membrane damages (**Figures 5E–G**). Observations made with SEM and TEM were consistent between each other, both showing morphological and ultrastructural changes restricted to a limited number of bacterial cells. Interestingly, food-grade E171 particles were found tightly associated to the cell surface, causing some deformation at the contact zone (**Figures 5H,I**).

### TiO_2_ Internalization Within *L. lactis* and *E. coli* Cells

**FIGURE 6:**
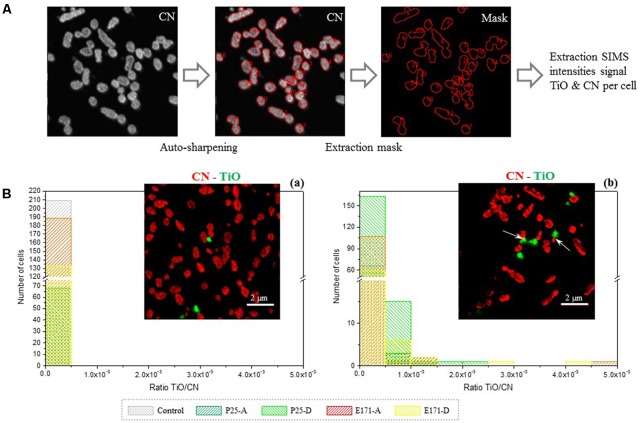
High-resolution nano-secondary ion mass spectrometry (Nano-SIMS) chemical imaging of bacterial cells after TiO_2_ exposure. **(A)** Methodology for the extraction of the SIMS intensities for each individual cell. From the SIMS CN image acquisition, by auto sharpening, each cell is individualized. The mask obtained is applied on image acquisition, allowing to extract the total SIMS intensities for each cell and for all the ions recorded; **(B)** Distribution of the TiO signal normalized by the CN signal among the bacterial population for **(a)** *L. lactis* IBB477 and **(b)** *E. coli* K12 MG1655 bacterial cells non-exposed (black hatched) and exposed to P25-A (dark green hatched), P25-D (light green hatched), E171-A (red hatched) or E171-D (yellow hatched). For each species a representative image is given with cells (in red) exposed to E171-A (in green) at 320 μg/mL. White arrows indicate internalized TiO_2_ particles. Samples were fixed, embedded and ultra-sectioned. Scale bar corresponds to 2 μm.

The conventional analytical capabilities associated with electron microscopy (EM), such as the Energy Dispersive Spectroscopy (EDS) or Electron Energy-Loss Spectroscopy (EELS), are not sensitive enough for the characterization of biological samples containing trace elements, with a detection limit of 0.1 at %. This explains why it was not possible to use EM to accurately assess whether or not TiO_2_ internalization occurred in bacterial cells. High-resolution Nano-SIMS was used, which combines high lateral resolution, high mass resolution and a high detection limit (few ppm per voxel) (Sangely et al., 2014; Höschen et al., 2015; Wirtz et al., 2015). The methodology developed and presented in **Figure 6A** allowed us to obtain semi-quantitative information at the subcellular scale (<100 nm) on the intracellular localization of Ti for *L. lactis* IBB477 and *E. coli* K12MG1655 exposed to E171 (E171-A and E171-D) or TiO_2_-NPs P25 (P25-A and P25-D). The intensity ratios between the trace element and the matrix element signals (TiO/CN) are displayed in **Figures 6Ba,b** for *L. lactis* and *E. coli*, respectively. The percentage of TiO/CN-positive bacterial cells is also given in **Table 3**. For *L. lactis* cells, no TiO_2_ internalization was observed (**Figure 6Ba** and **Table 3**). In contrast, for *E. coli*, about 7% of bacterial cells internalized TiO_2_ particles (**Table 3**), with the highest levels achieved with food-grade TiO_2_ (**Figure 6Bb**). This corresponds to the altered growth kinetics, and morphological and ultrastructural changes depicted above.

**Table 3:** Titanium dioxide internalization within *L. lactis* and *E. coli* cells as determined with high-resolution nano-secondary ion mass spectrometry (Nano-SIMS) chemical imaging.

	P25			E171		
	(P25-A+P25-D)			(E171-A+E171-D)		
		
	Total number of cells	Number of TiO/CN-positive cells	Percentage of TiO/CN-positive cells	Total number of cells	Number of TiO/CN-positive cells	Percentage of TiO/CN-positive cells
*L. lactis* IBB477	229	0	0	323	0	0
*E. coli* K12 MG1655	240	19	7.9%	186	13	7.0%


*The percentage of TiO/CN-positive cells is given for L. lactis IBB477 and E. coli K12 MG1655, and for TiO_*2*_-NPs P25 and food-grade E171 (sum of aggregated and dispersed forms)*.

## Discussion

In this study, Nano-SIMS- and synchrotron UV fluorescence-based imaging was used in combination with electron microscopy to provide valuable mechanistic insights into the toxicity of food-grade TiO_2_ (additive E171) to commensal intestinal and transient food-borne bacteria. The presence of a nano-sized fraction in this additive, as shown by Yang et al. (2014) and Dudefoi et al. (2017b) is increasingly suspected to play a role in disruption of intestinal homeostasis and development of gut microbiota dysbiosis. The microbiota, an important gut player, is rarely explored in food nanotoxicology (Fröhlich and Fröhlich, 2016; Fröhlich and Roblegg, 2016; Mercier-Bonin et al., 2016; Pietroiusti et al., 2016), apart from two recent studies which focus on E171 vs. P25 (Dudefoi et al., 2017a; Waller et al., 2017). Both studies were based on an *in vitro* colon model, inoculated with a gut microbial community from a healthy donor (Waller et al., 2017) or the defined anaerobic human gut bacterial community MET-1 (Dudefoi et al., 2017a). Depending on the conditions under study (source and characteristics of food-grade TiO_2_, concentration of TiO_2_ particles, exposure duration, model colon reactor design), different results were obtained. Food-grade TiO_2_ showed little impact on bacterial respiration, fatty acid profiles and phylogenetic composition (Dudefoi et al., 2017a); in contrast, changes to the microbial composition (i.e., inhibition of the control-induced shift in microbial composition from *Proteobacteria* to *Firmicutes* phyla) were found, together with some alterations of biochemical responses like a decrease in colonic pH (Waller et al., 2017). These differences in gut ecosystem functioning may be explained, at least to some extent, by direct interaction between TiO_2_ particles and bacteria (Jiang et al., 2009; Waller et al., 2017).

To address the question of TiO_2_/bacteria interactions and related toxicity, we selected a panel of Gram-negative/Gram-positive bacterial species from diverse origins and biotopes, including commensal and transient food-borne bacteria, exposed to non-UV-irradiated food-grade TiO_2_ (E171) vs. TiO_2_-NPs (P25). Apart from commensal bacteria, transient food-borne bacteria may also be in contact with TiO_2_ through ingested foods and/or during their passage through the gut, where they can also impact the resident microbiota and thus possibly the health of the host (Veiga et al., 2014; Derrien and van Hylckama Vlieg, 2015; Zhang et al., 2016). To date, the only study dealing with interactions between exogenously applied bacteria and nano-sized TiO_2_ relates to the food-borne pathogen *Listeria monocytogenes* (Ammendolia et al., 2014). In the present study, bacterial growth was inhibited by TiO_2_ in all selected bacteria (*E. coli*, *L. lactis*, *L. rhamnosus*, *L. sakei*, and *S. thermophilus*), particularly by food-grade TiO_2_. Based on these data, we focused on *E. coli* as a representative of Gram-negative commensal intestinal bacteria (Fröhlich and Fröhlich, 2016) and widely used as a model to characterize the antibacterial properties of nano-sized TiO_2_ (Liu et al., 2010; Kumar et al., 2011; Zhukova et al., 2012; Erdem et al., 2015; Sohm et al., 2015; Planchon et al., 2017). Indeed, *E. coli* is one of the first bacterial species to colonize the intestine and remains the predominant aerobic organism in the gastrointestinal tract into adulthood (Tenaillon et al., 2010). Additionally, we used the lactic acid bacterium *L. lactis* as a model of Gram-positive food-borne bacteria. The strain *L. lactis* IBB477 we studied possesses adhesive properties toward components of the intestinal mucosa (Radziwill-Bienkowska et al., 2014, 2016, 2017), which can be partly responsible for the prolonged and/or enhanced contact between these exogenously applied bacteria and the host.

In our study, imaging with DUV fluorescence was performed. Under these conditions, the photoluminescence of TiO_2_ particles enables tracking their accumulation in bacteria with a higher spatial resolution compared to conventional optical methods (Jamme et al., 2010). In previous works, this technique has been successfully employed to probe the distribution of antimicrobials in *Enterobacter aerogenes* (Kašèáková et al., 2012) as well as the accumulation of tryptophan-functionalized gold and silver nanoparticles in *E. coli* (Pajović et al., 2015; Dojčilović et al., 2016). Here, DUV fluorescence imaging with single-cell resolution revealed TiO_2_/cell co-localization by enabling to distinguish between the fluorescent signal pertaining to TiO_2_ particles and the autofluorescence of treated *E. coli* or *L. lactis* bacterial cells. As further shown by scanning and transmission electron microscopy, in a few cases, such TiO_2_/cell co-localization resulted in morphological and ultrastructural damages to both *L. lactis* and *E. coli*. One part of the bacterial population strongly interacted with food-grade E171 and to a lesser extent with TiO_2_-NPs P25 while the other part was totally free of TiO_2_ on the cell surface. This is completely in line with the findings of Planchon et al. (2017) on *E. coli* exposed to TiO_2_-NPs, which underlined that, which underlined that such heterogeneities, probably due to physiological variability, could account for the different metabolomic and proteomic responses observed.

To gain further insight into the mechanisms involved, the internalization of TiO_2_ in bacterial cells was probed using Nano-SIMS imaging. This method, initially developed for isotopic measurement at the single-cell level (Lechene et al., 2006; Li et al., 2008), was first applied to elemental silver-exposed *E. coli* (Saulou-Bérion et al., 2015). In fact, image acquisition at the subcellular scale (<100 nm) allowed for determination of the cell shape, thanks to the CN^-^ signal, and the intracellular localization of Ti (TiO^-^ signal). The ratio between intensities of the minor element TiO, and the major matrix ions CN, was measured per individual bacterium, which gives semi-quantitative values on the ability of the Ti element to enter the cells, for all experimental conditions tested. In contrast with *L. lactis*, for approximately 7% of *E. coli* cells exposed to food-grade TiO_2_, internalization occurred. These findings correspond to toxicity results of E171-exposed bacteria, where alterations in growth kinetics and viability reduction were greatest for *E. coli*.

This is the first *in vitro* study demonstrating the bacterial toxicity of food-grade TiO_2_, which differs from the P25 OECD reference nanomaterial, probably due to its specific physicochemical properties (Yang et al., 2014; Dudefoi et al., 2017b). Previous works under similar non-UV-irradiated conditions mainly focused on the effects of P25 or “P25-like” TiO_2_-NPs, generally tested in *E. coli*. Depending on the experimental conditions (size of TiO_2_-NPs, exposure regime), *E. coli* mortality was in the range between 20 and 80% (Erdem et al., 2015; Sohm et al., 2015; Planchon et al., 2017) with Gram-negative bacteria (*E. coli*) more susceptible than Gram-positive ones (*Bacillus subtilis*) (Erdem et al., 2015), which is fully consistent with our results. In other studies, morphological damages were depicted, together with probable internalization even though only qualitative observations were provided at this stage (Liu et al., 2010; Lin et al., 2014). TiO_2_-NPs also induced lipid peroxidation and disruption of cellular respiration (Erdem et al., 2015), membrane depolarization, loss of integrity and fluidity, and higher cell permeability (Liu et al., 2010; Sohm et al., 2015). These alterations on *E. coli* were further dissected at the transcriptomic, proteomic and metabolomic levels to identify biomarkers (Sohm et al., 2015; Planchon et al., 2017). Moreover, TiO_2_-NPs were found to induce both DNA damage and oxidative stress (Kumar et al., 2011), even though in the later study of Sohm et al. (2015), very few genes/proteins linked to oxidative stress were deregulated. Although beyond the scope of the present study, it would be valuable to further decipher the primary mode of action of food-grade TiO_2_ on *E. coli* and other commensal intestinal bacteria, as well as transient food-borne bacteria.

To conclude, our study provides new insights on interactions under non-UV irradiated conditions between TiO_2_ (food-grade E171 vs. TiO_2_-NPs P25) and bacteria of the gut microbiota (e.g., *E. coli*) or ingested with food (e.g., *L. lactis*) using innovative biophysics-based methods. We showed that TiO_2_ can be trapped by bacteria, which in turn induces moderate bacterial toxicity; *E. coli* exposed to food-grade TiO_2_ showed the most striking effects. However, especially during chronic exposure, the continuing presence of TiO_2_ within the intestine may reasonably be expected to produce substantial changes in the composition and the metabolic activity of microbiota, by its influence on the most sensitive bacteria. Furthermore, alterations in gut microbiota composition have been shown to occur in response to microbial challenges, variations in diet, and intestinal disorders. Deleterious effects of the food additive E171 might thus be potentiated by these factors. The TiO_2_-trapping capacity of food-borne bacteria could also be exploited to improve conditions for a healthy gut. All these areas of research should be further considered in risk assessment of dietary nanoparticles.

## Author Contributions

EH, PL, MT, and MM-B conceived and designed the study. JR-B, PT, VR, FJ, MR, MT, and MM-B performed and analyzed the Synchrotron SOLEIL experiments. CC, IF, and MM-B performed the electron microscopy experiments. EL and J-NA performed and analyzed the Nano-SIMS experiments. JR-B, JB, and MK chose the *L. lactis* IBB477 strain and culture conditions. MM-B, JK, JR-B, J-NA, EH, and MT wrote the manuscript. All authors contributed to the discussion and approved the final manuscript.

## Conflict of Interest Statement

The authors declare that the research was conducted in the absence of any commercial or financial relationships that could be construed as a potential conflict of interest.

## Footnotes

1 https://nano.bwh.harvard.edu/
