# Supplementary material for: Toxicity of Food-Grade TiO2 to Commensal Intestinal and Transient Food-Borne Bacteria: New Insights Using Nano-SIMS and Synchrotron UV Fluorescence Imaging
Source: Front Microbiol. 2018 Apr 24;9:794. doi: 10.3389/fmicb.2018.00794 (PMC5928251; doi:10.3389/fmicb.2018.00794)
Supplement: Supplementary file 1 [file Presentation_1.pdf]

Supplementary material

Supplementary material 1. Data analysis after Synchrotron excitation deep ultraviolet (DUV) fluorescence imaging.

A set of specific FIJI macro scripts has been written to standardize the analysis. First, visible light images were measured (mean, std dev and max values) to compute background value as mean value. Bacteria were assumed to be present in the areas, where the values exceeded the mean value (by 1.5 of std dev to max value). Default B&W threshold operation was run to receive 1bit input for "Particle Analysis" that resulted in finding individual bacteria as ROI data. For *L. lactis* 40-220 px area was taken while for *E. coli* 60-400 px, with circularity <0.1-1.0> in both cases. Single ROI selections were made as bacteria mask for each sequence. Further, the measurements (area, mean, std dev, modal, min, max, median, skewness, kurtosis) were made for tryptophan fluorescence (F3) and TiO_2 fluorescence (F6) images for all image area, all bacteria using previously obtained bacteria mask and background image area by inverting bacteria mask. Finally, TiO_2 fluorescence data corrected images (DC) were created in order to find TiO_2 particle condensation areas by subtracting bacteria fluorescence noise in adjusted F3 images from adjusted F6 images. F6 images data were adjusted by subtraction of background value (for *L. lactis* bg=815, for *E. coli* bg=790) and division by 60 (value depending on applied microscope filter). F3 images data were adjusted by subtraction of background value (for *L. lactis* and *E. coli* bg=659) and division by 26 (value depending on applied microscope filter). The co-localization of TiO_2 particles with bacteria was verified by merging the obtained DC images with visible images for bacteria.

Supplementary material 2. Statistical analysis of growth kinetics of *L. lactis* IBB477 and *E. coli* K12 MG1655 bacterial cells after exposure to TiO_2-NPs P25 and food-grade E171 in aggregated (A) and dispersed (D) forms with concentrations ranging from 32.0 to 320.0 µg/mL. Significance was determined using two-way ANOVA corrected for multiple comparisons with Bonferroni test: (*) $p<0.05$, (**) $p<0.01$ and (***) $p<0.001$ compared to control conditions with no TiO_2 added (ns: not significant). For sake of clarity, significance was not given in Figure 2 and is indicated below, in each condition and for different time points.

**Strains**	***L. lactis***				***E. coli***			
**TiO_2 (µg/mL)**	**P25-A**	**P25-D**	**E171-A**	**E171-D**	**P25-A**	**P25-D**	**E171-A**	**E171-D**
**32.0**	1-2h ***<br>3h **<br>4h and above ns	ns	1h ns<br>2h ***<br>3h *<br>4h and above ns	1h **<br>2-3h ***<br>4h and above ns	1-4h ns<br>6-14h ***<br>16h **<br>18-20h ns	1-2h ns<br>4h *<br>6-10h ***<br>12h **<br>14-20h ns	ns	ns
**62.5**	1h ns<br>2h ***<br>Above 2h ns	ns	1h ns<br>2h ***<br>3h and above ns	1-3h ***<br>4h and above ns	1-2h ns<br>4h *<br>6h and above ***	1-4h ns<br>6h *<br>8h **<br>10-16h ***<br>18-20h **	1-2h ns<br>4h and above ***	1-2h ns<br>4h and above ***
**125.0**	1h ns<br>2h ***<br>3h *<br>4h and above ns	1-5h ns<br>7h *<br>9-11h **<br>13h *<br>15h and above ns	1h *<br>2-3h ***<br>4h and above ns	1-2h ***<br>3h ns<br>4h *<br>5-11h ***<br>13-15h **<br>17-19h *<br>21h **	1-2h ns<br>4h and above ***	1-2h ns<br>4h and above ***	1-2h ns<br>4h **<br>6h and above ***	2h and above ***
**320.0**	1h ns<br>2h and above ***	1h ns<br>2h and above ***	1h *<br>2h and above ***	1h and above ***	1-2h ns<br>4h and above ***	2h and above ***	2h and above ***	2h and above ***
